# Disturbance and predation risk influence vigilance synchrony of black‐necked cranes *Grus nigricollis*, but not as strongly as expected

**DOI:** 10.1002/ece3.7196

**Published:** 2021-01-27

**Authors:** Dejun Kong, Anders Pape Møller, Yanyun Zhang

**Affiliations:** ^1^ Key Laboratory of Special Biological Resource Development and Utilization of Universities in Yunnan Province College of Agronomy and Life Sciences Kunming University Kunming China; ^2^ Ministry of Education Key Laboratory for Biodiversity Science and Ecological Engineering College of Life Sciences Beijing Normal University Beijing China; ^3^ AgroParisTech Ecologie Systématique et Evolution CNRS Université Paris‐Saclay Orsay France

**Keywords:** anthropogenic disturbance, collective vigilance, ecotourism, nature visitation, predation risk

## Abstract

Animals monitor surrounding dangers independently or cooperatively (synchronized and coordinated vigilance), with independent and synchronized scanning being prevalent. Coordinated vigilance, including unique sentinel behavior, is rare in nature, since it is time‐consuming and limited in terms of benefits. No evidence showed animals adopt alternative vigilance strategies during antipredation scanning yet. Considering the nonindependent nature of both synchronization and coordination, we assessed whether group members could keep alert synchronously or in a coordinated fashion under different circumstance. We studied how human behavior and species‐specific variables impacted individual and collective vigilance of globally threatened black‐necked cranes (*Grus nigricollis*) and explored behavior‐based wildlife management. We tested both predation risk (number of juveniles in group) and human disturbance (level and distance) effects on individual and collective antipredation vigilance of black‐necked crane families. Adults spent significantly more time (proportion and duration) on scanning than juveniles, and parents with juveniles behaved more vigilant. Both adults and juveniles increased time allocation and duration on vigilance with observer proximity. Deviation between observed and expected collective vigilance varied with disturbance and predation risk from zero, but not significantly so, indicating that an independent vigilance strategy was adopted by black‐necked crane couples. The birds showed synchronized vigilance in low disturbance areas, with fewer juveniles and far from observers; otherwise, they scanned in coordinated fashion. The collective vigilance, from synchronized to coordinated pattern, varied as a function of observer distance that helped us determine a safe distance of 403.75 m for the most vulnerable family groups with two juveniles. We argue that vigilance could constitute a prime indicator in behavior‐based species conservation, and we suggesting a safe distance of at least 400 m should be considered in future tourist management.

## Graphical abstract

Synchronization to coordinated vigilance pattern was first reported in a single bird species, Black‐necked crane *Grus nigricollis*. The bird showed synchronized vigilance in low disturbance areas, with fewer juveniles and far from observers; otherwise, they scanned in coordinated fashion. Vigilance could constitute a prime indicator in behavior‐based species conservation; a safe distance of at least 400 m was determined and should be considered in future tourist management.

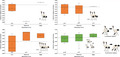

## 1 INTRODUCTION

Our nature is experiencing unprecedented changes, like dramatic extinction of species and decrease in abundance, which are commonly recognized as results of human‐driven global deterioration of ecosystems (IPBES, 2019). Anthropogenic disturbance on wildlife is not limited to human residential and nearby areas, but also penetrated into protected areas through nature‐based visitation and recreation (Geffroy et al., 2015; Marion et al., 2016; Slater et al., 2019). This threatens wildlife behavior, abundance, reproduction (Steven et al., 2011), and increases vulnerability of prey to predators (Geffroy et al., 2015). Conservationists believe that exploration of indicator behavior of wildlife may help us understand how wildlife experience anthropogenic impacts, and subsequently advance behavior‐based management, so as to achieve sustainable conservation and ecotourism (Berger‐Tal et al., 2011; Blumstein, 2017). One such indicator behavior is antipredator vigilance (Berger‐Tal et al., 2011).

Animals scan environments to monitor potential threats from both predators and rivals, regarded as vigilance (Beauchamp, 2015; Caro, 2005). Numerous studies of antipredator behavior revealed that animals gather in large groups to decrease individual vigilance, share collective vigilance benefits, and avoid being captured (Pulliam, 1973). A great deal of these studies focused on individual vigilance based on the assumption that an individual initiates vigilance bout regardless of behavioral state of others, termed independent vigilance (Caro, 2005; Pulliam, 1973). Cooperative vigilance, including synchronization and coordination in terms of behavior patterns, however, were also observed in multiple animal species when taking vigilance bouts between group companions into consideration (Pays, Jarman et al., 2007; Pays, Renaud et al., 2007).

Synchronized vigilance indicates individuals copying the state of vigilance in neighbors (Pulliam, 1973) leading to collective vigilance waves (Beauchamp, 2010), also known as allelomimetic vigilance (Pays, Jarman et al., 2007; Quenette & Gerard, 1992). Allelomimetic vigilance in groups may also induce collective waves of other activities, for example, foraging waves (Quenette & Gerard, 1992) and sleep waves (Beauchamp, 2010). Evidence from case studies and model analysis illustrated how group members could often synchronize their vigilance in the field (Rodríguez‐Gironés & Vásquez, 2002; Sirot & Touzalin, 2009).

While coordinated vigilance refers to group members remaining vigilant alternatively in order to avoid scan gaps of independent and synchronized vigilance by chance (Bednekoff, 2015). Sentinel behavior, a well‐known form of coordinated vigilance was observed to a limited extent in vertebrates with cooperative breeding behavior (Bednekoff, 2015), including mammals (Clutton‐Brock et al., 1999; Rasa, 1986), birds (Wickler, 1985; Wright et al., 2001), and fish (Brandl & Bellwood, 2015). Since the likely potential benefits in most cases are small, time payoff in coordinated vigilance would be less valuable (Ward, 1985) and only rarely occur in nature (Rodríguez‐Gironés & Vásquez, 2002).

Comparison with group vigilance when each individual scans independently, collective vigilance is named so as at least one group member is vigilant. This behavior should be expected to be more extreme in coordinated groups and less in synchronized groups (Pays, Jarman et al., 2007). So far, three vigilance strategies were documented, respectively, in different study systems and no omnibus vigilance strategy (two or more mixed vigilance of independent, coordinated, or synchronized) was reported yet in one species. Ge et al. (2011) reported synchrony of collective vigilance of paired red‐crowned crane *Grus japonesis* in family groups decreasing when birds shifted from the core zone with less disturbance to the buffer zone with higher disturbance. While the common crane *Grus grus* showed coordinated vigilance in the buffer zone which was considered as the first observation in birds (Ge et al., 2011). However, it is hard to tell whether the nonindependent vigilance of synchronization and coordination is species‐ or environment‐dependent. Basically, smaller common cranes showed stronger antipredator vigilance than larger crane species (Kong et al., 2020), which means that common cranes could adopt an elevated vigilance strategy to red‐crowned cranes facing similar threats or disturbances, for example, the buffer zone disturbance in the study of Ge et al. (2011). Thus, regardless of species, we could make a rational inference that crane pairs may decrease vigilance synchrony with increase in disturbance and shift to coordinated vigilance as predation risk or disturbance increase further in small groups (Wickler, 1985). So, we wondered whether synchronized and coordinated vigilance could be detected in nature, for a single species, concerning their common nature of nonindependency among members, and which factors drive the alteration between synchronization and coordination vigilance.

We examined the temporal pattern of nonindependent vigilance (synchronization and coordination) in a crane species, black‐necked Ccane *Grus nigricollis*, a species exclusively distributed on the plateau, a species that is an ideal candidate that could be easily observed at a distance for testing vigilance cooperation (Li et al., 2017). The birds were facing direct interference from nature‐based tourism and indirect threats from climate change and anthropogenic expansion, which induced loss of wetlands dramatically (Harris & Mirande, 2013; Li, 2014).

Previous studies documented synchronization vigilance decreasing between groups being driven by disturbance (Ge et al., 2011) and predation risk (Podgórski et al., 2016). Thus, coordinated collective vigilance patterns, as a stronger antipredation response against disturbance could be observed for birds under intense disturbance, while synchronized vigilance would be expected under lower levels of disturbance. We hypothesized that black‐necked cranes could decrease synchrony of vigilance with increase in disturbance and predation risk, even shift to coordinated vigilance, if possible. Since nature‐based recreation in habitats of black‐necked cranes is common, here we take both disturbance level (categorial variable) and observer distance (continuous variable) into consideration. If alternation in vigilance of synchronization to coordination could be detected, vigilance of paired black‐necked cranes would vary as a function of continuous variables of observer distance (*X*‐axis). The intercept value of the function with *X*‐axis at which paired cranes alter vigilance from synchronized to coordination, this could be regarded as a control (safe) distance keeping tourists away from the birds in order to lowest human interference. This control distance could be a valuable reference for future conservation aimed at management of tourists.

To sum up, in this study, we tested (1) how environmental and group variables affected both individual and collective vigilance of black‐necked cranes; (2) how they responded to nature‐based recreation through antipredator vigilance adjustment; and (3) what is the safety observation distance for both visits and scientific research purposes from the study of vigilance, which could benefit conservation and decision‐making.

## 2 MATERIALS AND METHODS

### 2.1 Study species

The black‐necked crane is a vulnerable species on the IUCN Red List, endemic to the western plateaus of China (including the Qinghai‐Tibet and Yunnan‐Guizhou Plateau) with <5% of the population inhabiting wetlands of Bhutan and India (Li, 2014). Breeding pairs occupy relatively permanent territories while nonbreeding subadults wander across vast areas in flocks (Zhang et al., 2017), aggregating in flocks or small family groups in winter (Liu et al., 2008). Family groups usually consist of parents and 0, 1, or 2 juveniles. It is easy to distinguish juveniles from adults from blocky blackish brown feathers, smaller body size, and whistle‐like calls (Kong, Zhong, Zhang et al., 2014). Adults have a distinct black neck, flight feathers, and tail compared with white plumage. However, telling males apart from females is hardly possible except from appearance of unison call (Kong, Zhong, Zhang et al., 2014).

### 2.2 Study area

**FIGURE 1:**
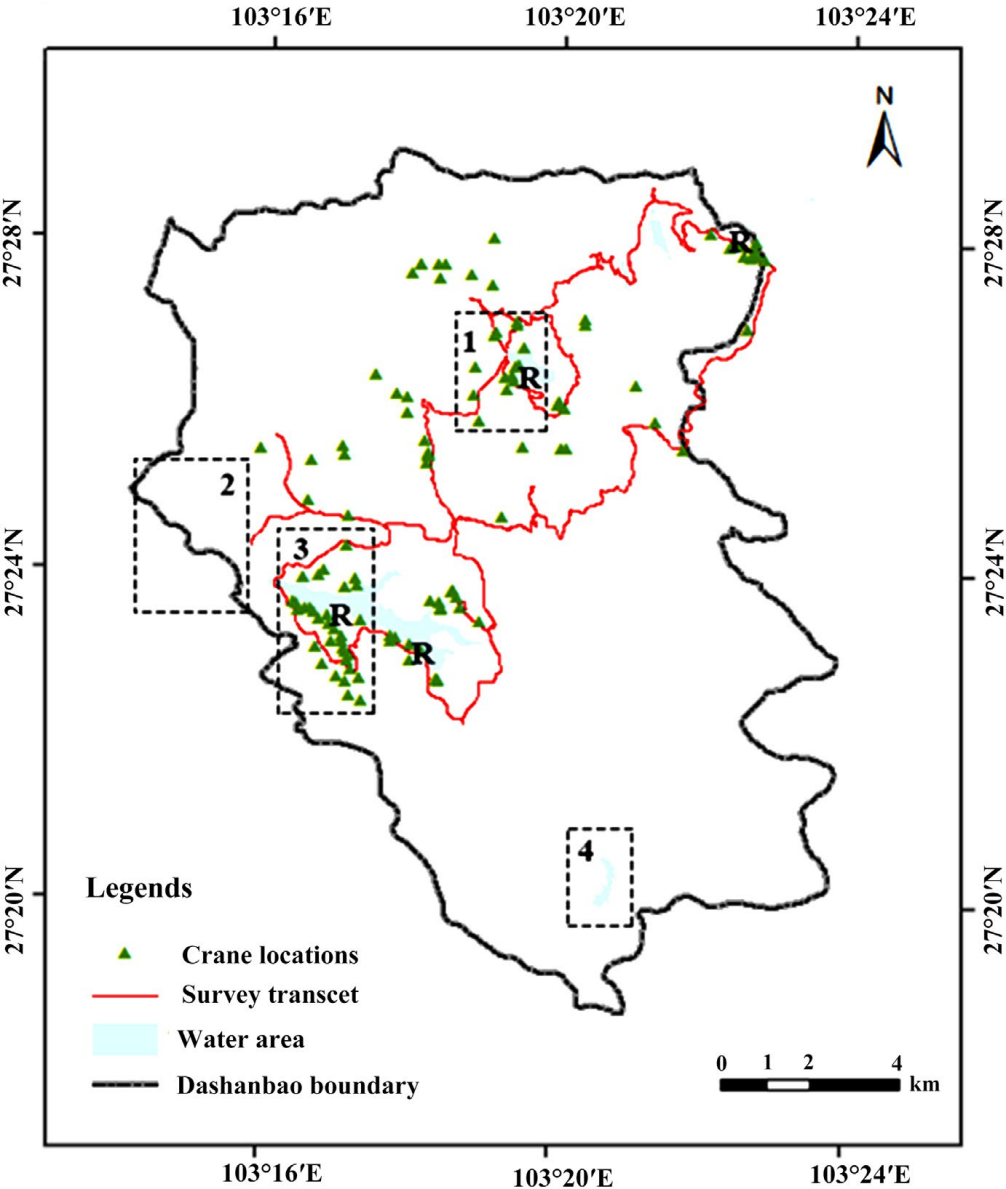
Study area of Dashanbao National Nature Reserve, Yunnan, SW China. The four dashed line regulars numbered 1–4 is the four areas visited by tourists named Dahaizi lake area, Jigong mountain and valley area, Tiaodunhe lake area, and Yanmaidi lake area, respectively. “R” indicates roosting sites. Noting that crane locations in this map only show the distribution of black‐necked cranes in our study area, but not the families locations taken into analysis in this study

This study was conducted in the Dashanbao National Nature Reserve (Dashanbao, N27°18′38″−27°29′15″, E103°14′55″–103°23′49″), SW China. Covering an area of 19,200 ha, Dashanbao was first established in 1993 and upgraded to national level in 2003 for protecting the globally threatened black‐necked crane and plateau wetlands on which waterfowl depend. Dashanbao was famous not only for its role as an important wintering ground for black‐necked cranes, but also a vital staging and stopover site for the eastern population of the bird (Kong, Zhong, Yang et al., 2014). Each year, there are nearly 1,500 individuals wintering at Dashanbao, constituting 40% of their eastern population and ~14% of the world population (Yang & Zhang, 2014). In 2004, Dashanbao was designated as a Ramsar wetland of international importance as its contribution in black‐necked crane conservation. There are four roosting sites, named Dahaizi, Xiaohaiba, Changhuikou, and Yinjiabeihaizi, located along the lakeside of Dashanbao reserve (Figure 1). Dahaizi is the most famous site as supporting the largest number of 600–900 black‐necked cranes each year (Kong, Zhong, Yang et al., 2014).

Gatherings of cranes attracted massive influx of people for bird‐watching, photography and landscape viewing. The local authority started a nature‐based tourism program in 2009. According to reports from the Dashanbao administration bureau, over 10,000 tourists, more than 10 times before tourism was implemented, went to Dashanbao at the end of 2009, with a yearly rate of increase of >30%. Over 60,000 people were recorded in 2013–2014 (Yang & Liu, 2014), sixty times increase in abundance after the public was allowed to enter. Sharp increase in tourism caused great threats to the black‐necked crane population and other wildlife. The Dashanbao ecotourism plan designated four discrete areas (named Dahaizi lake area, Tiaodunhe lake area, Jigong mountain and valley area, and Yanmaidi lake area) opened to the public, three of the four black‐necked roosting sites were located in two of these areas (Figure 1). People went to Dashanbao visit the Dahaizi Lake area (for crane observation and photograph) and Jigong mountain and valley area the most (Yang & Liu, 2014), occasionally they drove around the Tiaodun Lake, but seldom to Yanmaidi lake where cranes were occasionally present (Peng et al., 2020). Thus, cranes distributed in the Dahaizi and Jigong area and along the roadside to these areas are facing high levels of disturbance, and cranes in Tiaodunhe area facing intermediate disturbance while cranes in the other areas encountered the lowest disturbance (Figure 1). There are no natural predators for black‐necked cranes, except for direct or indirect interferences from human beings and occasional mortality from anthropological infrastructures (e.g., powerlines) and domestic dogs.

### 2.3 Data collection

We conducted behavioral observations of black‐necked cranes during 09:00–18:00 during the winter 2013–2014. Cranes were firstly detected along a 96‐km long line transect by vehicle (Figure 1). Generally, we checked the line transect every 7–10 days in clear days without fog, snow, or rain. We changed the direction in two surveys next to each other, and cranes detected along the transect were only recorded once the same day so as to reduce the risk of repeated sampling. As no birds were banded in our study, which posed a risk of repetition, but the probability is negligible due to the large population there. Once cranes were located one observer walked to the focal flock and collected behavioral data of the birds with a digital video recorder (Sony HDR‐XR260E with 55 × optical zoom) set on a tripod. As rare shelter (e.g., trees or shrubs) could be found in the mosaic landscape of farmland, grassland, and marsh, cranes could detect our approach at a large distance, we stopped approach as long as we could distinguish their behavior on the screen of the video recorder. We kept our body down to the ground as low as possible for 3 min (mins) to reduce the alert response of the cranes, then started the behavioral sampling. We kept still during videotaping. Meanwhile distance to the birds was measured with a laser rangefinder monocular (Newcon Optik LRM 2200SI with a maximum range of 2,200 meters). We aimed to take 10 min film for each family group encountered, because it is sufficiently long to get sufficient vigilance bouts for black‐necked cranes (Li et al., 2017). Sometimes we got longer taping if the focal birds did not encounter disturbance so as to meet our sampling criteria of 10 min, since unexpected scarcity to cranes happened frequently. In order to get time series data of each family member, we made sure each individual was in the picture at the same time; film recording ceased when one bird was out of sight or flew away (Ge et al., 2011). Vigilant and nonvigilant behavior was extracted in seconds (s) from videos taken in the field. We defined head‐up scanning with neck stretched while standing, walking or even laying down as vigilance (Kong et al., 2020), otherwise as nonvigilant behavior.

### 2.4 Data processes

All our filmed sequences were sufficiently stable to distinguish each behavioral state and long enough to meet at least 5 samples of vigilance/nonvigilance bouts for statistical effectiveness, even for our shortest video sampling of 2.2 min, as scan and interscan duration for black‐necked crane are 4s and 20s on average (Li et al., 2017). Thus, all our data were entered in the subsequent analysis. Vigilance behavior (individual vigilance) was evaluated as both the proportion of vigilance and duration for each group member. Vigilance proportion was calculated as percentage of time spent on vigilance during the observation secession, while vigilance duration as the average time span (second) of each vigilance bout (Li et al., 2017). Collective vigilance was regarded as at least one bird keep vigilant in the group (Ge et al., 2011), we only take adults into consideration when calculating group collective vigilance as no vigilance interactions between juveniles and adults were detected (Che et al., 2018; Ge et al., 2011). Group collective vigilance was calculated as percentage of time with at least one adult with head up. We determined collective vigilance of coordination and synchronization by vigilance spectral analysis, time pattern or time series of two adults in family groups (Pays, Jarman et al., 2007).

We classified the areas where cranes occurred into low, moderate, and high disturbance levels according to tourist accessibility. We considered cranes distributed in the scenery spots of Dahaizi Lake and Jigong mountain‐valley area and along the road between them facing the highest disturbance since nearly 90% tourists to Dashanbao visited these places. Cranes were under moderate disturbance in the areas around Tiaodunhe Lake with <30% of public visitation, and lowest disturbance in the other areas where tourists seldomly visited (Yang & Liu, 2014). We considered the observer as proxy of potential tourists, thus the distance from observer to cranes is another effect variable on vigilance. Because inexperience always correlated with high predation vulnerability, a higher proportion of juveniles in groups usually indicated higher predation risk (Beauchamp, 2015; Xu et al., 2013). We considered our study objectives of three family types with two adults and 0–2 juveniles as three levels of predation risk. Adults without juveniles endure the lowest predation risk while families with two juveniles have the highest predation risk.

Since our data deviated from normality in a one‐way Kolmogorov–Smirnov test, and arcsine square root transformation for vigilance proportion and logarithmic transformation for vigilance duration were subsequently conducted in order to get normalized data for parametric tests. Comparisons of vigilance difference between adults and juveniles and observed to expected collective vigilance of two adults were accomplished with *t* test. One‐way ANOVA was used to test both individual and collective vigilance difference of adults in three family types and under different disturbance level. We selected linear mixed models to distinguish effects of disturbance level (categorical variable of three levels), observer distance (continuous variable), and predation risk (categorical variable of three family types) on both individual vigilance of black‐necked crane, with family ID as a random factor (Ge et al., 2011). Collective vigilance deviation was used to determine collective vigilance pattern (synchrony or coordination) by considering the deviation between expected and observed collective vigilance. Coordinated vigilance occurs when collective vigilance deviation above the value of zero, otherwise synchronized vigilance occurs (Ge et al., 2011; Pays, Jarman et al., 2007). Expected collective vigilance was calculated with the equation of 1–[(1–*p_1_*)*(1–*p_2_*)], where *p* represents vigilance proportion of two adults in a family (Ge et al., 2011; Pays, Jarman et al., 2007). Person correlation analysis was used to explore observer distance effect on collective vigilance deviation, the difference between observed and expected collective vigilance. Statistics were accomplished with IBM SPSS 20.0 software with a two‐tailed significant level of <0.05.

## 3 RESULTS

### 3.1 Individual vigilance

In total, we obtained 870 min of observations from 34 family groups, including 6 families without juvenile, 17 families with one juvenile, and 11 families with two juveniles. For each family the observation lasted for 10.2 min on average (range: 2.2–23.5 min), which met our expectation of 10 min.

Adults spent significantly more time on vigilance (adults: 27.30%–31.28% versus juveniles: 8.74%–16.65%; *p* < .05) and relatively longer vigilant bout (adults: 12.05–12.81s versus juveniles: 8.42–11.78s; *p* > .05) than juveniles (Table 1). No significant difference in adult vigilance proportion (*F* = 0.106, *df* = 2, *p* = .899) or duration (*F* = 0.833, *df* = 2, *p* = .440) among three family groups existed.

**TABLE 1:** Vigilance (proportion and duration) difference between adults and juveniles in three family groups of *Grus nigricollis*

Family types	Age	Vigilance proportion		Vigilance duration/Second	
		Mean ± *SD*	*t* test	Mean ± *SD*	*t* test
F2−0 (*n* = 6)	Adults (*n* = 12)	28.29 ± 25.16%	**—**	17.36 ± 29.53	—
F2−1 (*n* = 17)*	Adults (*n* = 33)	27.30 ± 20.00%	*t* = 2.212, *df* = 47, ***p* = .032**	12.05 ± 14.34	*t* = −0.039, *df* = 47, *p* = .969
	Juveniles (*n* = 16)	16.65 ± 16.69%		11.78 ± 10.81	
F2−2 (*n* = 11)*	Adults (*n* = 21)	31.28 ± 21.45%	*t* = 4.354, *df* = 32.024, ***p* = .000**	12.81 ± 10.28	*t* = 1.471, *df* = 36, *p* = .150
	Juveniles (*n* = 20)	8.74 ± 8.72		8.42 ± 5.69	

Note

The first figure for each family type is the number of adults and the latter is the number of juveniles in a family group. Bold figures indicate significant level of *p* < .05. Occasionally, not all the members of a family group could be sampled at the same time (n indicates sample size), and one adult and one juvenile were missed in F2‐1 group while one adult and two juveniles were missed in F2‐2 group as indicated with * symbol in the table.

We detected significant observer (distance) effect on vigilance behavior of adults (vigilance proportion: *F*_24, 37_ = 3.130, *p* = .001; vigilance duration: *F*_24, 36_ = 1.891, *p* = .041) and juveniles (vigilance duration: *F*_19, 10.092_ = 3.073, *p* = .036).

### 3.2 Collective vigilance and synchrony

**FIGURE 2:**
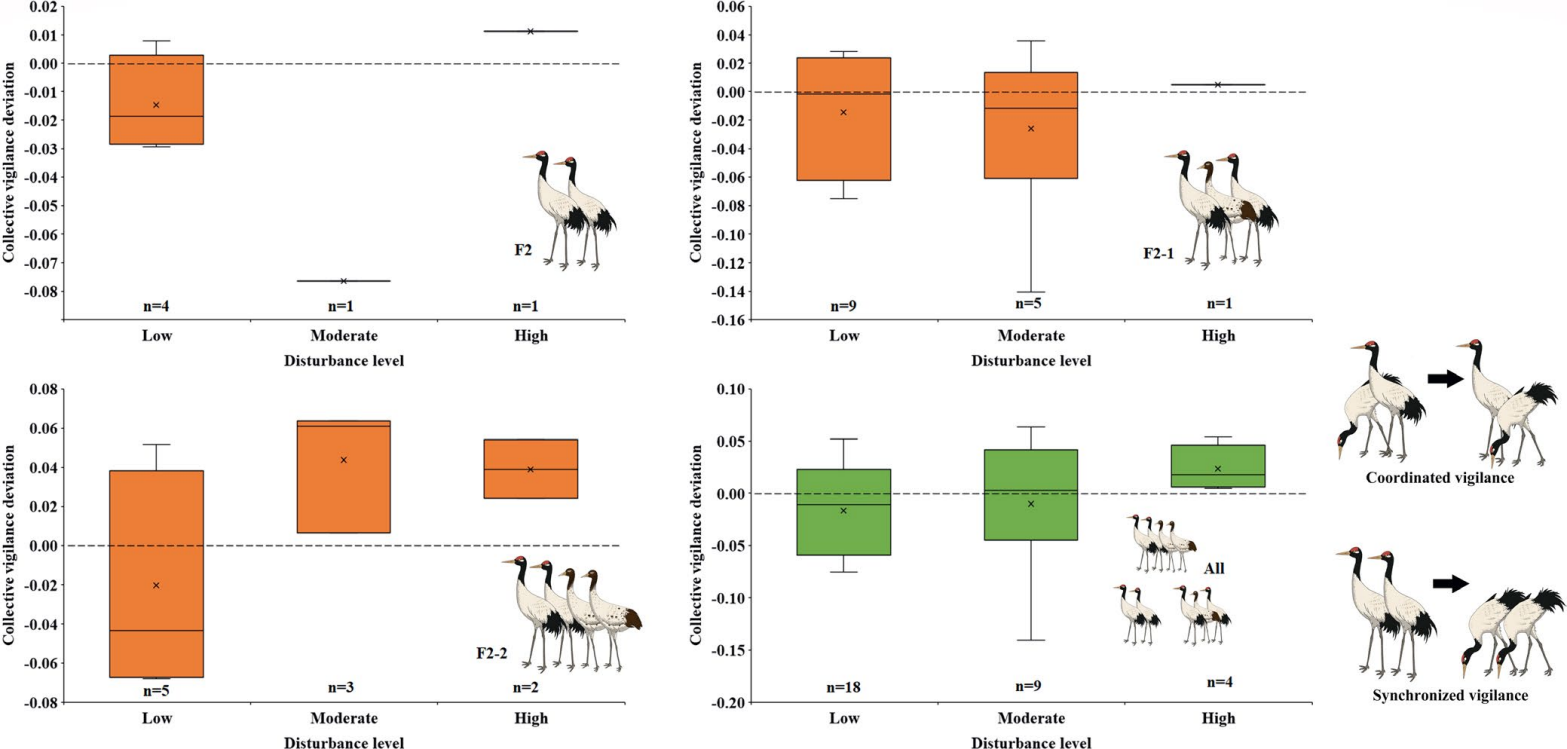
Comparison of observed and expected collective vigilance of two adults in three family groups. The figure “2” in group composition indicates two adults in a family group and the latter figure “0–2” indicates the number of juveniles in a family. Positive value of collective vigilance deviation means coordinated vigilance while negative is synchronized vigilance. n indicates sample size. The box plots show mean (symbol *x*), median, quartiles and 5 and 95 percentiles

**FIGURE 3:**
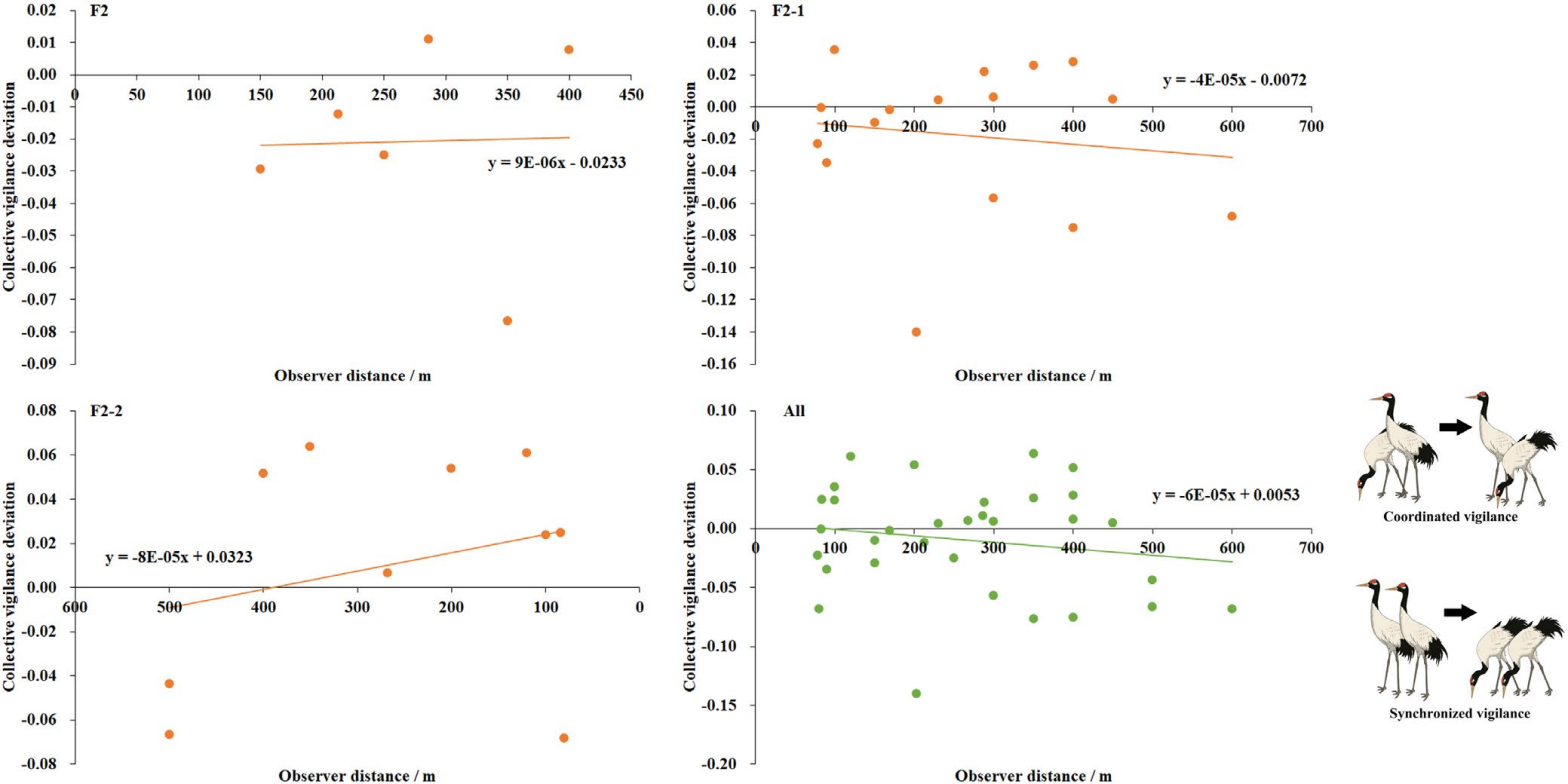
Collective vigilance function of two adults in family groups to observer distance. Positive value of collective vigilance deviation means coordinated vigilance while negative is synchronized vigilance. F2‐0, F2‐1, and F2‐2 indicate family groups of two adults with 0–2 juveniles

We found, in family groups, adults without juveniles behaved synchronized collective vigilance in low disturbance areas, with disturbance increase and more juveniles present, they behaved vigilant in a coordinated pattern (Figure 2). Crane couples with 0–1 juvenile scan in synchrony constantly, while couples with 2 juveniles scan from synchronization to coordination pattern at 403.75 m as a human was approaching (Figure 3).

However, the collective vigilance observed did not differ from expectation for all three family types under different disturbance levels, did not vary significantly from each other (*F* = 0.593, *df* = 2, *p* = .559); and the collective deviation did not correlate with observer distance significantly either (Table 2).

**TABLE 2:** Comparison of observed and expected collective vigilance (with paired *t* test analysis) for two adults in a family group under different disturbance level

	Family types		
	F2‐0 (*n* = 6)	F2‐1 (*n* = 16)	F2‐2 (*n* = 10)
Collective vigilance			
Observed	42.29 ± 29.81%	43.96 ± 24.60%	54.59 ± 28.78%
Expected	44.36 ± 31.79%	45.73 ± 24.01%	53.51 ± 24.87%
Disturbance level (Paired *t* test)			
Low	*t* = −1.763, *df* = 3, *p* = .176	*t* = −1.063, *df* = 8, *p* = .319	*t* = −0.821, *df* = 4, *p* = .458
Moderate	—	*t* = −1.050, *df* = 5, *p* = .342	*t* = 2.341, *df* = 2, *p* = .144
High	—	—	*t* = 2.605, *df* = 1, *p* = .233
Observer distance (Person correlation)	*r* = 0.027, *p* = .960	*r* = −0.130, *p* = .632	*r* = −0.268, *p* = .454

Note

Person correlation analysis was used to explore observer distance effect on collective vigilance deviation (difference between observed and expected collective vigilance). Note that not all 34 observed family groups were taken into consideration as one adult was missed in one F2‐1 and one F2‐2 family, making calculation of collective was impossible. Thus, sample size in collective vigilance analysis is 32.

## 4 DISCUSSION

### 4.1 Collective vigilance synchrony

With spectral analysis of vigilance behavior that takes time sequences of vigilant and nonvigilant state into consideration, we reported the first evidence of group vigilance shift from synchronized to coordinated pattern for a single species. The vigilance synchronization was related to disturbance and predation risk, but no significant effects were detected, revealing black‐necked crane couples scan the environment independently as reported in former studies (Che et al., 2018).

Although our results indicated no significant difference between observed and expected collective vigilance, the obvious trends from synchronized to coordinated vigilance with increase in disturbance (level and observer proximity, Figures 2 and 3) and predation risk (number of juveniles) showing clearly that coordination vigilance occurs in situations of rather high threats or disturbance, as both theoretical (Rodríguez‐Gironés & Vásquez, 2002) and case studies have demonstrated (Bednekoff, 2015; Ge et al., 2011; Wickler, 1985; Wright et al., 2001).

Compared with a similar study on crane vigilance, Ge et al. (2011) first reported common crane were vigilant coordinately in buffer zones with time expense of nearly 40% (individual vigilance; collective vigilance: 57.7%), and the sympatric red‐crowned crane behaved with synchronization vigilance with time expense of <20% (individual vigilance; collective vigilance: 32.8%), while black‐necked cranes (individual vigilance: ~30%; collective vigilance: 42.3%–54.6%) in our study showed insignificant coordination/synchronization vigilance (or independent vigilance). That is because black‐necked cranes did not face so high disturbance as common cranes or so little disturbance as red‐crowned cranes did. Another explanation is that our sample size was still small considering three family groups and three disturbance levels (Figure 2).

Coordination is time‐costly (Ward, 1985) and sentinels are at great risk of getting depredated (Ridley et al., 2013), making this behavior evolutionarily unstable (Rodríguez‐Gironés & Vásquez, 2002; Sirot & Touzalin, 2009). Thus, groups only select this strategy under high risky situations (Ge et al., 2011). Although coordination recognized as a kind of time‐waste strategy, it ensures more scanning coverage and fewer alert gaps for foraging groups (Pulliam, 1973; Sirot & Touzalin, 2009).

### 4.2 Factors influencing collective vigilance

Many studies demonstrated that cooperative sentinel activity (one form of coordinated vigilance) increases when pups are present (Santema & Clutton‐Brock, 2013) and when predation risk becomes greater (Rauber & Manser, 2017; Ridley et al., 2010). These findings support ours that cranes kept vigilant in a coordinated pattern, when more young birds are present, in high disturbance areas and when humans show proximity. In our study, we considered the number of juveniles in a family group as predation vulnerable or at risk (Xu et al., 2013), as predators prefer hunting those left behind or inexperienced individuals (Sirot & Touzalin, 2009). Thus, more juveniles suffer costs and more adults are involved in collective alert time as our study demonstrated (Figure 2).

### 4.3 Antipredation strategy of Black‐necked cranes

Our study confirmed former studies (Che et al., 2018; Wang et al., 2009) that adults took the responsibility of antipredator scanning with significantly higher alert time expenditure and duration than juveniles. Wildlife decreased their individual vigilance and benefitted from foraging by gathering in big flocks (Kong et al., 2020; Pulliam, 1973), but intraspecific conflicts also increased with group size (Caro, 2005; Pulliam, 1973), and juveniles were more vulnerable to conflicts. So, in winter, we could observe many family groups (2 adults with 1–2 juveniles) foraging separately from other large groups (Liu et al., 2008), so as to keep >80% of time foraging for young birds (Wang et al., 2009). Adults in family groups have to spend ~6% more time than adults in large groups in vigilance instead of foraging (Wang et al., 2009). Interestingly, in order to avoid high predation risk faced by family groups, both sequence organization and interscan intervals of individual vigilance of black‐necked cranes are randomly distributed (Li et al., 2017, 2018), making vigilance bouts more unpredictable than predators could initiate successful attack.

### 4.4 Individual vigilance across winter grounds and increased with tourism development

Compared with other wintering grounds of black‐necked cranes in China, including those at Caohai wetlands in Guizhou Province (individual vigilance: 12.59%–16.52%; Li & Ma, 1992), Napahai wetlands in Yunnan (individual vigilance: 11.74%–17.05%) and Lhasa in Tibet (individual vigilance: 17.8%–21.0%; Che et al., 2018), black‐necked cranes in our study area spent most time allocated to vigilance (27.30%–31.28%). Black‐necked cranes in our study area (Dashanbao) are facing equivalent disturbance to the birds in Daqiao wetland (individual vigilance: 27.9%; Kong et al., 2020) with a large human density of 135 residents per square kilometers, about 80km away from Dashanbao. Similarly, individual vigilance of cranes in our study and in Daqiao is significantly affected by human disturbance (Kong et al., 2020). The predominant landscape of vast marshes in Napahai and Caohai wetlands contributed to the avoidance of human access to crane habitats resulting in lower levels of antipredator scans. While wintering habitats utilized by cranes in Dashanbao and Daqiao are farmland (Kong et al., 2011, Kong et al., 2018), making the cranes avoid human disturbance impossible.

From a historical point of view, black‐necked cranes in this study (~30% of time allocation) spent even more time on vigilance than former research conducted here in 2008 (individual vigilance: 15.6%–21.4%; Kong et al., 2008), when ecotourism was not carried out yet. Our study also demonstrated that the explosive expansion of nature‐based tourism caused new threats to threatened black‐necked cranes (Li, 2014).

### 4.5 Conservation and management implications

Collective vigilance of two adults varied in a linear function with observer distance, which helps us determine a distance at which cranes change vigilance from synchronized to coordinated behavior. According to our results, a distance of 88.33 m and 403.75 m for black‐necked crane population and the most vulnerable family with two juveniles was determined, respectively. The safe distance of 88.33 m at population level is very close to the flight initiation distance (FID, the distance at which birds take flight to approaching human and is generally used as a quantitative measure of a bird's tolerance to human‐caused disturbance; Møller et al., 2019) of 76 m for the birds (our unpublished data conducted at the same place), indicating high reliability and repeatability of our results. A study on observer effect on vigilance in red‐crowned cranes reported that birds spent the lowest time in vigilance when the observer was 400 m away (Li, 2013). Considering that animals also benefited from large group size (Pays, Jarman et al., 2007), the 2‐2 family actually was the most vulnerable group for black‐necked cranes. Thus, we suggest to keep tourists at least 400 m away from black‐necked cranes so as to minimize disturbance from human beings, from species conservation and ecotourism management viewpoints.

## CONFLICT OF INTEREST

All authors declare that they have no competing interests.

## AUTHOR CONTRIBUTION

**Dejun Kong:** Conceptualization (lead); Data curation (lead); Formal analysis (lead); Funding acquisition (lead); Investigation (lead); Methodology (lead); Project administration (lead); Resources (lead); Supervision (lead); Validation (lead); Writing‐original draft (lead); Writing‐review & editing (lead). **Anders Pape Møller:** Writing‐original draft (supporting); Writing‐review & editing (supporting). **Yan‐Yun Zhang:** Formal analysis (supporting); Software (lead); Writing‐original draft (supporting); Writing‐review & editing (supporting).

## Data Availability

All datasets in the present study are available at Dryad https://doi.org/10.5061/dryad.5mkkwh73j
